# Trouble with the curve: the 90–9-1 rule to measure volitional participation inequalities among Royal Canadian Mounted Police cadets during training

**DOI:** 10.3389/fpsyt.2024.1297953

**Published:** 2024-05-28

**Authors:** Taylor A. Teckchandani, Robyn E. Shields, Katie L. Andrews, Kirby Q. Maguire, Laleh Jamshidi, Jolan Nisbet, Tracie O. Afifi, Lisa M. Lix, Sherry H. Stewart, Shannon Sauer-Zavala, Rachel L. Krakauer, J. Patrick Neary, Gregory P. Krätzig, R. Nicholas Carleton

**Affiliations:** ^1^Canadian Institute for Public Safety Research and Treatment-Institut Canadien de recherche et de traitement en sécurité publique (CIPSRT-ICRTSP), University of Regina/Université de Regina, Regina, SK, Canada; ^2^Anxiety and Illness Behaviors Lab, Department of Psychology, University of Regina, Regina, SK, Canada; ^3^Department of Community Health Sciences, University of Manitoba, Winnipeg, MB, Canada; ^4^Department of Psychology, Dalhousie University, Halifax, NS, Canada; ^5^Department of Psychology, University of Kentucky, Lexington, KY, United States; ^6^Faculty of Kinesiology & Health Studies, University of Regina, Regina, SK, Canada; ^7^Department of Psychology, University of Regina, Regina, SK, Canada

**Keywords:** mental health, RCMP cadets, public safety personnel, volition, participation

## Abstract

**Objective:**

The Royal Canadian Mounted Police (RCMP) Study includes longitudinal multimodal assessments of RCMP cadets from pre-training (i.e., starting the Cadet Training Program [CTP]) to post-deployment and for five years thereafter. The data allow for investigating the multidimensionality of volitional participation in digital health data collection frameworks within serial data collection platforms and the impact of participation inequalities by classifying cadets using the 90–9-1 rule. By classifying cadets as Lurkers, Contributors, and Superusers formally described by the 90–9-1 rule, where 90% of actors do not participate, 9% seldom contribute, and 1% contribute substantially allows for the assessing of relationships between participation inequalities in self-monitoring behaviors as well as whether mental health disorder symptoms at pre-training (i.e., starting the CTP) were associated with subsequent participation.

**Methods:**

Participants were asked to complete a Full Assessment prior to their training at CTP, as well as short daily surveys throughout their training. Participation frequency was described using a process where participants were rank ordered by the number of daily surveys completed and classified into one of three categories. Full assessment surveys completed prior to their training at CTP included screening tools for generalized anxiety disorder (GAD), major depressive disorder (MDD), posttraumatic stress disorder (PTSD), alcohol use disorder (AUD), and panic disorder (PD). The Kruskal-Wallis H test was used to assess differences in participation rates between mental health disorder symptom screening groups for each measure at pre-training, and Spearman’s Rho was used to test for associations amongst self-reported Full Assessment screening tool responses and the number of daily surveys completed during CTP.

**Results:**

There were 18557 daily survey records collected from 772 participants. The rank-ordering of cadets by the number of daily surveys completed produced three categories in line with the 90–9-1 rule: Superusers who were the top 1% of cadets (*n*=8) and produced 6.4% of all recordings; Contributors who were the next 9% of cadets (*n*=68) and produced 49.2% of the recordings; and Lurkers who were the next 90% of cadets (*n*=695) and produced 44.4% of daily survey recordings. Lurkers had the largest proportion of positive screens for self-reported mental health disorders at pre-training.

**Conclusion:**

The creation of highly individualized, population-based mental health injury programs has been limited by an incomplete understanding of the causal relationships between protective factors and mental health. Disproportionate rates of disengagement from persons who screen positive for mental health disorders further compounds the difficulty in understanding the relationships between training programs and mental health. The current results suggest persons with mental health challenges may be less likely to engage in some forms of proactive mental health training. The current results also provide useful information about participation, adherence, and engagement that can be used to inform evidence-based paradigm shifts in health-related data collection in occupational populations.

## 1 Introduction

Public safety personnel (PSP) include, but are not limited to, border services personnel, correctional workers, firefighters, paramedics, police officers, and search and rescue personnel (1). PSP are frequently exposed to potentially psychologically traumatic events (PPTE) as a function of their occupational duties (2). PPTE include direct or indirect exposure to actual or threatened death, serious injury, or sexual violence (1). Exposures to PPTE are associated with increased posttraumatic stress injuries (PTSIs; e.g., major depressive disorder [MDD]; posttraumatic stress disorder [PTSD]) among PSP (3–7). The Royal Canadian Mounted Police (RCMP) report frequent and diverse PPTE exposures (2); in addition, up to half of serving RCMP officers may screened positively for one or more mental health disorders, including PTSD (30.0%), MDD (31.7%), generalized anxiety disorder (GAD; 23.3%0; social anxiety disorder (SAD; 18.7%), panic disorder (PD; 12.0%), and alcohol use disorder (AUD; 3.9%) (4). Early identification of a PTSI could substantially improve prognosis and improve RCMP wellbeing (8).

The RCMP Study (9) provides an opportunity to assess for relationships between completion rates of daily surveys (i.e., regular mental health monitoring) and mental health status. Relative to serving officers, cadets starting the Cadet Training Program (CTP; i.e., pre-training) report far fewer symptoms consistent with mental health disorders (i.e., 2.7% PTSD, 6.6% MDD, 3.6% SAD, 1.6% PD, 0.0% AUD, and 11% GAD; 9). RCMP Study participants volitionally complete short (i.e., ~60 seconds) daily surveys (i.e., assessing mood, attitude, performance, physical wellness, emotional state, hours worked, hours slept, quality of sleep, eating patterns, social activity, physical activity, substance use). The daily surveys allow for ongoing self-monitoring of mental health status. The extant literature suggests an inverse relationship between self-monitoring of mood and self-reported mental health disorder symptoms (8). Self-monitoring of mood can enhance emotional self-awareness and self-regulation (10, 11), thereby increasing help-seeking behaviors (8, 12). Participation analyses are most often used for implementation evaluations associated with digital social health networks, health related blogs, and internet phenomenon (13, 14); however, analyzing daily survey participation may also identify relationships between participation patterns and mental health, informing potential options for providing better supports.

The quantitative evaluation of participation inequalities has emerged as a crucial topic in the successful implementation and management of digital health platforms (15–20). The relationships between participant motivation, use, engagement, and actor status have identified participation inequalities mirroring the 80–20 Pareto principle and participation patterns such as the 90–9-1 rule (18, 21, 22). The 90–9-1 rule classifies user participation into three categories; specifically, Lurkers, Contributors, and Superusers, where 90% of actors do not participate, 9% seldom contribute, and 1% contribute significantly to the content (15, 17, 18, 22, 23).

The current study was designed to examine the relationship between volitional participation in daily surveys (i.e., the quantity of within-participant daily survey recordings collected during the 26-week CTP) and screening positive for one or more mental health disorders based on self-reported symptoms at pre-training. The current study will also assess for multidimensionality of volitional participation within digital health data collection frameworks and the serial data collection platforms used in the RCMP Study (9). The 80–20 distribution and the 90–9-1 Rule have been applied to biomedical and digital health networks, but the current application is a novel test of RCMP Study participation inequalities associated with groupwise differences across superusers, contributors, and lurkers (9, 17–19, 24). Cadets who screened positively for one or more mental health disorders at pre-training (i.e., starting the CTP) were expected to have fewer recordings than cadets who did not screen positively because of the inverse relationship observed between changes in mental health disorder symptom scores and self-monitoring in RCMP cadets (25).

## 2 Materials and methods

### 2.1 Procedure

The current study used data from the RCMP Study, which has been described in detail within a published dedicated protocol paper (i.e., 9). The RCMP Study was approved by the University of Regina Institutional Research Ethics Board (file No. 2019–055) and the RCMP Research Ethics Board (file No. SKM_C30818021312580). The RCMP Study was also approved through a Privacy Impact Assessment as part of the overall approval including the National Administrative Records Management System (NARMS; file No. 201611123286) and Public Services and Procurement Canada (PSPC; file No. 201701491/M7594174191). Study data were collected via online self-report surveys. Mental health disorder symptom self-report surveys were collected at pre-training (i.e., when starting the CTP) and daily surveys were collected throughout the CTP.

### 2.2 Sample and data

Participants for the current study were RCMP cadets (*n* = 772; 72% male) who completed the 26-week CTP as part of the Standard Training Program (9). The current study inclusion criteria required participants at pre-training to have completed all items on the administered mental health disorder screening tools administered at pre-training Cadets were Canadian citizens or permanent residents, 19 to 57 years old, who can fluently read, write, and speak either English or French (26). Cadets must meet several recruiting requirements, including security clearances, medical examinations, a polygraph test, and minimum physical standards. There were no conditions requiring exclusion of persons otherwise qualified for the CTP. Participants were provided smartphones free of charge, to facilitate data collection and participation as needed. All communications between the research team and participants, as well as the administration of surveys and individual participant feedback were coordinated through a tailored and dedicated, Protected B status instance of the online learning platform Moodle, paired with an app downloaded to compliant smartphones and accessed using a secured Qualtrics account. Data transfers from participant devices to secured Protected B status research servers in Canada were protected using Transport Layer Security. The RCMP Study also employs a PKI Class 3 SSL Certificate, with a 2048-bit digital signature and 256-bit encryption.

### 2.3 Self-report measures

Self-report mental health screening tools were administered online at pre-training and included the PTSD Check List 5 (PCL-5; 27); the 9-item Patient Health Questionnaire (PHQ-9; 28); the 7-item Panic Disorders Symptoms Severity Scale, Self-Report (PDSS-SR; 29); the 7-item Generalized Anxiety Disorder scale (GAD-7; 30); the 14-item Social Interaction Phobia Scale (SIPS; 31); and the 10-item Alcohol Use Disorders Identification Test (AUDIT; 32). Questionnaire descriptions and psychometric properties have been provided in the dedicated protocol paper (i.e., 9).

For the PCL-5, per the Diagnostic and Statistical Manual of Mental Disorders, 5th edition (DSM-5; 33), participants reported on their lifetime exposures (i.e., exposures prior to attending the CTP) to a specific list of PPTEs provided by the Life Events Checklist for the DSM-5 (LEC-5; 27, 34–36). The LEC-5 does not include “sudden and unexpected death of someone close to you” as a potential index PPTE (35). Participants select an index PPTE (i.e., “Consider which event from the list was the worst, most distressing event. If more than one of these events happened to you, select the one event that currently causes you the most distress”) against which to rate their past month symptoms using the PCL-5 items. A positive screen on the PCL-5 required participants to report exposure to at least one LEC-5 item, meet minimum criteria for each PTSD cluster, and have a total score >32 (27).

PHQ-9 and GAD-7 items were reported on for the previous 14 days, PDSS-SR items for the previous 7 days, SIPS items for no specified time frame, and AUDIT items for the past 12-months. Based on published guidelines for total scores, positive screens for each scale were established: PHQ-9 > 9 (37); PDSS-SR > 7 (38); GAD-7 > 9 (39); SIPS > 20 (31); and AUDIT > 15 (40). Measures have been validated for screening to identify people who may require follow-up with a clinician.

The daily surveys were brief 20-item self-report surveys completed via smartphone, taking approximately 60 seconds to complete. The daily surveys asked participants to report on the previous 24-hour period. The daily surveys assess participants on several domains including mood, attitude, performance, physical wellness, emotional state, hours worked, hours slept, quality of sleep, eating patterns, social activity, physical activity, and substance use, with details provided in the dedicated protocol paper (i.e., 9).

### 2.4 Sociodemographic variables

Sociodemographic characteristics were collected for each participant including sex and gender (i.e., male and female), age (i.e., 19 to 29 years, 30 to 39 years, 40 to 49 years, and 50 to 59 years), marital status (i.e., single, separated/divorced, and married/common-law), province of residence (i.e., Western Canada [British Columbia, Alberta, Saskatchewan, Manitoba], Eastern Canada [Ontario, Quebec], Atlantic Canada [Newfoundland & Labrador, Prince Edward Island, Nova Scotia, New Brunswick], or Northern Territories [Yukon, Northwest Territories, Nunavut]), and highest level of education (i.e., high school graduate or less, some post-secondary school, and university degree/4-year college or higher) (9).

### 2.5 Participation measures

Participation was measured as: (1) the number of completed daily survey recordings completed during CTP; and (2) rank-ordering of participation, in which cadets were rank-ordered by the number of daily surveys completed during their time at CTP and classified into one of three 90–9-1 categories. Superusers were the most frequent contributors to the daily surveys (i.e., top 1%), Contributors were the next most frequent contributors (i.e., next 9%), and Lurkers were the next most frequent contributors (i.e., next 90%).

### 2.6 Statistical analyses

Sociodemographic characteristics of study participants were described using frequencies and percentages. Percentages were used to describe the prevalence of positive screenings for each mental disorder at pre-training. To test for differences in the number of daily surveys across demographic groups, *t*-tests were used where there were participants in only two groups and analysis of variance (ANOVA) were used where there were participants in three or more groups. The Holm-Bonferroni procedure was used to control the familywise error rate to the nominal α = .05 for multiple tests. Effect size estimates for two group comparisons used Cohen’s *d* values (i.e., small, *d*=.20; medium, *d*=.50; large, *d*=.80) (41) and for more than two groups used partial eta squared ( $\eta_{p}^{2}$ ) (i.e., small, $\eta_{p}^{2}$ =.01; medium, $\eta_{p}^{2}$ =.06; large, $\eta_{p}^{2}$ =.14) (41).

The nonparametric Spearman’s rho coefficient was calculated to describe the relationship between the number of daily surveys completed and mental health disorder symptom scores at pre-training. Spearman correlations were calculated for the number of daily surveys completed by each cadet and their respective self-report mental health disorder symptom scores at the aggregate and screening category levels (i.e., PCL-5 total scores for all cadets; PCL-5 total scores for cadets that screened negative; PCL-5 total scores for cadets that screened positive) to test for linear relationships within screening categories. The Holm-Bonferroni procedure was implemented to adjust the significance threshold of correlation analyses to reduce the risk of type I errors from multiple comparisons.

The Kruskal-Wallis H test was used to assess differences in participation rates between mental health disorder symptom screening groups for each measure at pre-training (i.e., PCL-5, PHQ-9, SIPS, PDSS, GAD-7 and AUDIT). Shapiro-Wilke tests were performed to test for departures from normality for the mental health disorder symptom scores for the full sample. Complete or 100% participation was defined as at least one record per day for the full duration of CTP, up to a maximum of 182 records over the 26-week CTP. No duplicate recordings were observed. A detailed analysis of attrition, as well as the demographic profiles and pre-training mental health disorder symptoms can be found elsewhere (Carleton et al., submitted^1^). All values were compiled using Microsoft Excel (Microsoft Corporation, Seattle, USA) and imported to IBM SPSS Statistical Analysis Software (IBM, v.26 Premium, New York, USA) for statistical analyses.

## 3 Results

### 3.1 Sociodemographics

Details of self-reported participant demographics and symptom scores are provided in **Table 1**. Shapiro-Wilke tests indicated that no mental health disorder symptom data distributions departed from normality; accordingly, parametric statistical tests were used to compare mental health disorder symptom scores between sociodemographic categories. Participants were mostly male (72.0%), between the age of 19 to 29 years old (59.8%), and single (47.2%) or married/common-law (42.9%). Participants were mainly from Western Canada (52.8%) and reported having either some post-secondary school (43.4%) or a university degree, 4-year College or higher level of education (39.5%). All participants self-identified as cis-gendered, so only sex was used for the analysis.

**Table 1:** Participant Demographic and Mental Disorder Screening Measure Characteristics.

	Full Survey Sample^2^	PTSD (PCL-5)	*n*	MDD (PHQ-9)	*n*	Generalized Anxiety Disorder (GAD-7)	*n*	SAD (SIPS)	*n*	PD (PDSS-SR)^4^	*n*	AUD (AUDIT)	*n*
	*% (n)*	*Mean (SD)*		*Mean (SD)*		*Mean (SD)*		*Mean (SD)*		*Mean (SD)*		*Mean (SD)*	
Total Sample													
	100 (772)	5.95 (9.33)	697	3.19 (3.60)	762	4.17 (4.24)	767	5.22 (6.62)	768	4.45 (3.62)	78	3.64 (2.43)	612
Sex													
Male	72.0 (556)	5.36 (8.67)	498	2.93 (3.29)	550	3.77 (3.93)	555	4.74 (6.11)	554	4.79 (3.75)	33	3.81 (2.5)	435
Female	25.1 (194)	7.59 (10.79)	178	4.09 (4.35)	190	5.11 (4.75)	192	6.47 (7.49)	192	5.63 (3.00)	30	3.24 (2.22)	160
Test Statistic^1^	–	*t*(674)=-2.75**	–	*t*(738)=-3.84***	–	*t*(743)=-3.83***	–	*t*(744)=-3.19**	*-*	*t*(61)=-0.98	–	*t*(593)=2.52**	–
Effect Size (Cohen’s *d*)	–	0.240		0.323		0.321		0.267		0.248		0.233	
Age Group													
19–29 years	59.8 (462)	5.91 (9.29)	413	3.33 (3.75)	458	4.22 (4.29)	460	5.39 (6.64)	463	5.23 (3.84)	39	3.81 (2.53)	370
30–39 years	28.0 (216)	6.25 (9.31)	201	3.31 (3.58)	211	4.38 (4.26)	214	5.52 (6.79)	217	5.50 (2.86)	20	3.43 (2.18)	169
40–49 years	6.3 (49)	6.19 (11.12)	43	2.63 (2.95)	48	3.19 (3.29)	48	3.85 (5.07)	48	^	^	2.67 (1.94)	43
50–59 years	0.6 (5)	^	^	2.40 (1.52)	5	2.40 (2.07)	5	1.40 (2.61)	5	–	–	^	^
Test Statistic^1^	–	*F*(3,657)=0.48		*F*(3,718)=0.64		*F*(3,728)=1.36		*F*(3,724)=1.47		*F*(2,60)=0.09	–	*F*(3,581)=3.54*	
Effect Size ( $\eta_{p}^{2}$ )	–	.002		.003		.006		.006		.003		.018	
Marital Status													
Single	47.2 (364)	6.35 (9.62)	328	3.37 (3.78)	358	4.14 (4.08)	362	5.73 (6.78)	362	4.49 (3.85)	35	4.00 (2.63)^a^	280
Separated/Divorced	1.6 (12)	8.17 (9.39)	12	3.00 (2.63)	12	5.92 (4.64)	12	4.42 (4.60)	12	–	–	4.30 (2.00)^a,b^	10
Married/Common-Law	42.9 (331)	5.59 (9.06)	300	3.08 (3.55)	327	3.97 (4.24)	328	4.75 (6.29)	329	4.88 (2.94)	26	3.28 (2.17)^b^	264
Test Statistic^1^	–	*F*(2,637)=0.84		*F*(2,694)=0.54		*F*(2,699)=1.31		*F*(2,700)=2.03		*F*(2,59)=0.35		*F*(2,551)=6.46**	
Effect Size ( $\eta_{p}^{2}$ )	–	.003		.002		.004		.006		.012		.023	
Province of Residence^a^													
Western Canada (BC, AB, SK, MB)	52.8 (408)	6.63 (9.96)	373	3.43 (3.73)	403	4.62 (4.47)^a^	406	5.73 (6.63)	406	4.49 (3.50)	43	3.56 (2.26)	325
Eastern Canada (ON, QC)	34.6 (267)	4.50 (7.66)	240	2.70 (3.36)	263	3.34 (3.73)^b^	266	4.42 (5.83)	266	3.75 (3.80)	24	3.76 (2.67)	203
Atlantic Canada (PEI, NS, NB, NFL)	11.3 (87)	7.15 (10.52)	75	3.79 (3.69)	86	4.84 (4.38)^a,b^	85	5.48 (8.66)	86	6.10 (3.63)	10	3.80 (2.49)	75
Northern Territories (YK, NWT, NVT)	1.0 (8)	6.14 (9.74)	7	1.38 (0.92)	8	1.50 (1.41)^a,b^	8	2.38 (2.56)	8	–	–	2.43 (1.27)	7
Test Statistic^1^	–	*F*(3,691)=3.04*		*F*(3,756)=3.72**		*F*(3,761)=6.85***		*F*(3,762)=2.61*		*F*(2,74)=1.50		*F*(3,606)=0.99	
Effect Size $\;\left(\eta_{p}^{2}\right)$	–	.013		.015		.026		.010		.039		.005	
Education													
High school graduate or less	10.2 (79)	6.94 (8.79)	66	3.85 (4.21)	78	4.71 (4.62)	78	5.94 (7.56)	78	6.11 (3.33)	9	3.28 (1.94)	66
Some post-secondary school	43.4 (335)	5.92 (9.32)	309	3.29 (3.57)	328	4.15 (4.28)	332	5.07 (6.41)	332	5.38 (4.13)	21	3.84 (2.61)	262
University degree/4-year college or higher	39.5 (305)	5.71 (9.40)	271	3.05 (3.56)	303	3.97 (4.02)	304	5.20 (6.44)	305	4.87 (3.14)	30	3.56 (2.37)	246
Test Statistic^1^	–	*F*(2,643)=0.46		*F*(2,706)=1.56		*F*(2,711)=0.95		*F*(2,712)=0.55		*F*(2,57)=0.46	–	*F*(2,571)=1.65	
Effect Size ( $\eta_{p}^{2}$ )	–	.001		.004		.003		.002		.016		.006	

PTSD, post-traumatic stress disorder; PCL-5, PTSD Checklist for DSM-5; PHQ-9, Patient Health Questionnaire; GAD, Generalized Anxiety Disorder; SIPS, Social Interaction Phobia Scale; PDSS-SR, Panic Disorder Symptoms Severity Scale, Self-Report; AUDIT, Alcohol Use Disorders Identification Test.

aAB, Alberta; BC, British Columbia; MB, Manitoba; NB, New Brunswick; NFL, Newfoundland and Labrador; NS, Nova Scotia; NVT, Nunavut; NWT, Northwest Territories; ON, Ontario; PEI, Prince Edward Island; QC, Quebec; SK, Saskatchewan; YK, Yukon.

^1^The test results comparing scores on mental disorder screening measures for demographic variables are reported as t(degrees of freedom)=test statistic for g = 2 groups and as F(numerator degrees of freedom, denominator degrees of freedom)=test statistic for g> 2 groups.

^2^Total percentages may not sum to 100 and ns may not sum to 772 due to non-response or “other” responses.

^4^A limited number of participants reported values for PD (PDSS-SR) because selecting “No” for “ever having experience with panic attacks” or “having panic attack in the last 7 days”, meant participants were not presented the rest of the PDSS-SR questions.

^*^p<.05, ^**^p<.01, ^***^p<.001 – Statistically significantly different.^Sample size between 1 and 5, so data not presented. “-“ No data available.

Lettered superscripts within each column category indicate statistically significant differences between category groups with different letters on outcome at *p* ≤ .05.

### 3.2 Daily survey participation

Kruskal-Wallis tests indicated no statistically significant differences in the quantity of daily surveys completed by participants in screening groups on the AUDIT, *H*(1) = 1.247, *p* = .264, $\left(\eta_{p}^{2}\right)$ =.001. There were no statistically significant differences in the quantity of daily survey recordings between screening groups on the PDSS-SR, *H*(1) = 2.633, *p* = .268 $\left(\eta_{p}^{2}\right)$ =.001. There were no statistically significant differences in the quantity of daily survey recordings among the negative, mild, moderate, and severe GAD symptom screening groups on the GAD-7 questionnaire, *H*(1) = 0.112, *p* = .738, $\left(\eta_{p}^{2}\right)$ =.006. There were no statistically significant differences in the quantity of daily survey recordings among the negative, moderate, and severe MDD symptom screening groups on the PHQ-9, *H*(2) = 1.711, *p* = .425, $\left(\eta_{p}^{2}\right)$ =.002. There were no statistically significant differences in the quantity of daily survey recordings between the positive and negative screening groups on the PCL-5, *H*(1) = 1.247, *p* = .264, $\left(\eta_{p}^{2}\right)$ =.001.

Bivariate nonparametric within participant correlations indicated a statistically significant inverse relationship between pre-training PCL-5 total scores and the number of daily surveys completed at an aggregate sample level (*p*<.05), but there were no other statistically significant relationships between the number of recordings and the total scores at pre-training. Additionally, no statistically significant linear relationships were observed in pre-training self-report mental health disorder symptom scores amongst screening categories (**Table 2**).

**Table 2:** Mental Disorder Screening Prevalence (%) and Descriptive Statistics.

Mental Health Disorder Symptom Scale	*Screening Categories*	*% (n)*	*Record Count*	*Score Range*	*Median Participation % (IQR)*	*Rho*
PTSD (PCL-5)	Total Sample	100 (697)	18557	0-80	13.2 (25.0)	-.103*
	Negative	97.3 (678)	18143	0-32	13.2 (25.3)	-.040
	Positive	2.7 (19)	414	33-80	21.4 (22.5)	-.202
MDD (PHQ-9)	Total Sample	100 (762)	18557	0-27	13.2 (25.0)	-.040
	Negative	93.4 (712)	18420	0-14	13.7 (24.7)	-.028
	Moderate	6.2 (49)	127	15-19	9.9 (20.9)	-.389
	Severe	^ (^)	10	19-27	^	^
GAD (GAD-7)	Total Sample	100 (767)	18557	0-21	13.2 (25.0)	-.063
	Negative	(683)	12884	0-4	13.7 (25.8)	-.010
	Mild	(43)	4258	5-10	12.4 (25.3)	-.066
	Moderate	(29)	1040	11-15	14.8 (23.1)	-.118
	Severe	(12)	375	16-21	10.2 (19.2)	-.061
SAD (SIPS)	Total Sample	100 (768)	18557	0-56	13.2 (25.0)	-.025
	Negative	96.4 (740)	14997	0-33	13.1 (24.0)	.011
	Positive	3.6 (28)	3560	34-56	10.7 (25.3)	.291
PD (PDSS-SR)^1^	Total Sample	100 (772)	18557	0-28	13.2 (25.0)	-.067
	Negative	98.4 (760)	15246	0-11	13.1 (24.5)	.042
	Positive	1.6 (12)	403	12-28	9.0 (17.4)	-.185
Alcohol Use Disorder (AUDIT)	Total Sample	0.0 (0)	18557	0-16	13.2 (25.0)	.060

PTSD, posttraumatic stress disorder; PCL-5, PTSD Checklist for DSM-5; PHQ-9, Patient Health Questionnaire; GAD-7, Generalized Anxiety Disorder Scale; SIPS, Social Interaction Phobia Scale; PDSS-SR, Panic Disorder Symptoms Severity Scale, Self-Report; AUD, Alcohol Use Disorder; AUDIT, Alcohol Use Disorders Identification Test; IQR, Interquartile Range.

^*^p<.05 – Statistically significantly different.

^Sample size between 1 and 5, so data are not presented to protect participant anonymity.

^1^A limited number of participants reported values for PD (PDSS-SR) because selecting “No” for “ever having experience with panic attacks” or “having panic attack in the last 7 days”, meant participants were not presented the rest of the PDSS-SR questions.

### 3.3 Participation classifications

The participants designated as “Superusers” (*n*=8; i.e., top 1%) contributed 6.4% of all daily survey recordings during the CTP and all screened negative for PTSD, MDD, GAD, SAD, PD, and alcohol use disorder at pre-training (**Table 3**). The participants designated as “Contributors” (*n*=69; i.e., next 9%) were mutually exclusive from the Superusers and contributed to 49.2% of all daily survey recordings during the CTP. All Contributors screened negative for PD and alcohol use disorder at pre-training, and fewer than 5 screened positive for PTSD, SAD, or MDD (**Table 3**). There were 26 (37.7%) Contributors who screened positive for GAD (**Table 3**). The participants designated as “Lurkers” (*n*=695; i.e., next 90%) were mutually exclusive from the Superusers and Contributors and contributed 44.4% of all daily survey records collected during the CTP. The most positive screens at pre-training were among the Lurkers, with 31 (4.5%) screening positive for PTSD, 49 (7.1%) for MDD, 58 (8.3%) for GAD, 28 (4.0%) for SAD, and 12 (1.7%) for PD (**Table 3**).

**Table 3:** Mental Health Disorder Screening Prevalence and Demographics for 90–9-1 Participation Percentile Groupings.

Participation Classification	Current RCMP Study Cadet Participant Results at Pre-Training Assessment		
	Superusers: Top 1%	Contributors: Next 9%	Lurkers: Bottom 90%
Number of Records Produced	6.4% (988)	49.2% (7578)	44.4% (6834)
Disorder	% (*n*)	% (*n*)	% (*n*)
Alcohol Use Disorder – Past 12 Months			
Negative	0.0 (0)	0.0 (0)	0.0 (0)
Positive	0.0 (0)	0.0 (0)	0.0 (0)
PTSD (PCL-5)			
Negative	100 (8)	98.6 (68)	95.5 (664)
Positive	0.0 (0)	^	4.5 (31)
MDD (PHQ-9)			
Negative	100 (8)	98.6 (68)	92.8 (645)
Moderate	0.0 (0)	^	7.1 (49)
Severe	0.0 (0)	0.0 (0)	^
Generalized Anxiety Disorder (GAD-7)			
Negative	100 (8)	62.3 (43)	91.7 (637)
Mild	0.0 (0)	27.5 (19)	3.5 (24)
Moderate	0.0 (0)	^	3.5 (24)
Severe	0.0 (0)	^	1.4 (10)
SAD (SIPS)			
Negative	100 (8)	97.1 (67)	96.0 (667)
Positive	0.0 (0)	^	4.0 (28)
PD (PDSS-SR)^1^			
Negative	100 (8)	100 (69)	98.3 (683)
Positive	0.0 (0)	0.0 (0)	1.7 (12)

AUDIT, Alcohol Use Disorders Identification Test; GAD-7, Generalized Anxiety Disorder Scale; HAMOPD, History of Mood, Anxiety, and Other Psychiatric Diagnoses; PCL-5, PTSD Checklist for DSM-5; PDSS-SR, Panic Disorder Symptoms Severity Scale, Self-Report; PHQ-9, Patient Health Questionnaire; SIPS, Social Interaction Phobia Scale.

^Sample size between 1 and 5, so data are not presented to protect participant anonymity.

## 4 Discussion

Participating cadets were able to volitionally complete quick (i.e., ∼1 minute) daily surveys with self-assessments as part of the RCMP Study (9). The daily surveys allowed participants to track their physical and mental well-being. Cadets were encouraged to reflect on their emotions, physical health, emotional well-being, amount and quality of sleep, physical exercise, and drug use. The current study was designed to assess for associations between volitional participation inequalities in daily mental health monitoring and pre-training mental health, and subsequently demonstrates the potential for a predisposition to engage in self-monitoring behaviors based on pre-existing self-reported mental health disorder symptoms. Cadets who completed the most daily surveys during the CTP had fewest positive screens for mental health disorders, although the exact relationships are complex and nonlinear.

When interpreting potential confounding factors that contribute to nonresponse bias in the context of participation bias among Superusers, Contributors, and Lurkers within our RCMP Cadet sample, the relationships between pre-training mental health and daily survey participation differ across categories in both strength and direction in ways consistent with previously identified participation biases (15–20). Cadets in the current study also interact with and access the self-monitoring resources and digital social health tools in the same patterns as the general population (17, 18, 42, 43). No statistically significant groupwise differences were observed in the quantity of records produced at the categorical screening level, but groupwise differences were heavily biased by the Contributors and Lurkers (**Table 3**). Superusers (*n*=8; i.e., top 1% of Cadets) disproportionately contributed 6.4% of all daily survey recordings and screened negative at pre-training for PTSD, MDD, GAD, SAD, PD, and alcohol use disorder as measured by the self-report surveys. Contributors (*n*=69; i.e., the next 9% of Cadets) produced 49.2% of all daily survey recordings and most screened negative for most disorders. Lurkers (*n*=695; i.e., the next 90% of Cadets) produced the remaining 44.4% of all daily survey recordings but included the largest number of cadets who screened positive for one or more mental health disorders at pre-training. The results indicate groupwise differences in volitional participation distributions that skew groupwise total scores, and highlight the importance of identifying Superusers, Contributors, and Lurkers with measures of inequality over time, as daily self-monitoring requires consistent, voluntary participation to be therapeutic or prophylactic for mental health disorder symptoms, especially considering that 88.6% of participating RCMP cadets completed fewer than 50% of the possible daily surveys during CTP.

Research and treatment programs that recognize mental health is on a continuum and value promoting well-being may help to minimize stigma and increase help-seeking (7, 44–46). Self-monitoring is an important part of several scientifically validated psychological therapeutic techniques, including dialectical behavior therapy (47), acceptance and commitment therapy (48), Cognitive Behavioral Therapy (CBT; 45, 49, 50), and mindfulness training (10). Patients who record their own thoughts, feelings, and behaviors can analyze their reports as part of a CBT-based intervention and practice self-monitoring (11, 50, 51).

Poor emotional awareness is also a latent cause factor for symptoms of mood- and anxiety-related disorders (52, 53). The ability to identify and appreciate one’s own emotions is considered a crucial step in emotional self-awareness that has been positively correlated with adaptive control of emotions and improved mental health (54, 55). Mood self-monitoring can improve emotional self-regulation by increasing general emotional self-awareness (10, 11). By increasing emotional self-awareness and therefore emotional self-regulation through self-monitoring (48, 56), maladaptive anxiety responses can also be restructured, depression-perpetuating behaviors can be challenged (12), and PTSD patients can be well treated in a small but significant percentage of cases (51, 57). Results in the literature demonstrate a positive relationship between self-awareness and self-help behaviors, with a potential link between the process of self-monitoring supported by daily survey completion and therapy methods similar to CBT (12, 45, 49, 50, 58).

The multidimensional dynamicism of volitional participation in digital health frameworks and self-monitoring behaviors has been highlighted regarding digital social health network engagement (13, 16–20) and military applications (59–61). For example, in a cohort of 576,502 newly enlisted United States Military service members between the years of 2003 and 2006, cadets who had a mental health diagnosis at initial eligibility were 77% less likely to deploy and were at higher risk of early attrition (60). Therefore, the likelihood of deployment was considered lower and the risk of early attrition higher for persons with mental health diagnoses at pre-training (60). In the context of the RCMP Study, active engagement with survey material may increase cadet self-awareness and self-reflection, which may impact psychological processes mediating associations between daily survey participation and extant self-reported mental health disorder symptoms (12, 48, 50, 57). Accounting for a link between daily survey participation and self-reported mental health disorder symptoms at pre-training is crucial for subsequent studies assessing patterns of participation, mental health, and attrition, and his evidenced in a related study (25). There may be a voluntary participation bias, such that the non-response bias related to daily survey completions is associated with mental health resiliency, social support, or personality, and interacts with perceived barriers to help-seeking. The current study results highlight the potential factor of pre-existing mental health disorder status influencing engagement in self-monitoring behaviors, which increases the complexity of successfully implementing regularly administered measurement-based care in occupational and clinical settings.

Evidence-based self-monitoring may implicitly encourage meta-cognitive practices, support active engagement with positive choices for mental health, and facilitate earlier access to care. Accordingly, self-monitoring itself may be an under-used and readily -accessible intervention, in addition to being a tool for measurement-based care or evaluating intervention effectiveness within clinical trials (62). The current results require replication and extension; in the interim, the results provide useful information about participation, adherence, and engagement with self-monitoring, which may inform ongoing assessments of self-monitoring as a proactive intervention for protecting mental health.

### 4.1 Strengths and limitations

There are several strengths to the current study. First, a large quantity of records was collected from cadets who were recruited to the RCMP Study. The CTP environment facilitates serial data collection and promotes the measurement of participation, adherence, compliance, and attrition by following participants for up to 5 years after completion of the CTP. The classification of cadets using pre-screening self-report surveys facilitates the assessment of volitional engagement without the financial barriers that may otherwise restrict participation because participating cadets were provided with smartphones free of charge, as needed. Participation data collection provides useful information from which to build injury models in retrospect. Participation classification schema allows researchers to investigate the presence or development of changes in volitional engagement and participation that may manifest in the wake of a PTSI. Type I and Type II error risks were protected against by *a priori* statements of expected outcomes (9), and statistical corrections for multiple comparisons, respectively.

There are several limitations to the current study that inform directions for future research. First, there is a lack of data about cadets prior to their pre-screening self-report surveys upon entering the RCMP Study. This left censorship bias is managed by performing a series of evaluations to identify predispositions or underlying mental health disorders. Second, cadets with increased reporting of mental health disorder symptoms may have left the RCMP Study or the CTP because of having worse mental health, creating a self-selection bias within the collected data. Details regarding the causes of participant attrition are limited, with most participants who left the RCMP Study reporting having had insufficient time to participate. Lastly, the replicability and generalizability of the results to a general community sample using a digital health platform is limited by the structural and procedural facilitation of serial data collection during the CTP.

### 4.2 Future directions

Future directions include the use of survival analyses based on categorical screening variables to examine attrition at defined timepoints, as well as the median time to attrition between groups as data collection continues over the next 60 months. Logistic regression models with discriminant function analyses could also be performed to determine variables that contribute to group identity, considering the participation inequalities identified in the current paper. The 90–9-1 Rule should be implemented longitudinally to assess for changes in participation inequalities as cadets progress through their careers, with recalculations of the 90–9-1 rule participation categories to supplement inequality measures for self-monitoring interventions. The classification schema will allow researchers to assess for changes in volitional engagement and participation that may manifest after a PTSI, whether the injury occurs before a cadet enters the CTP or during field deployment. Effectively examining engagement trends can reciprocally enhance adherence through occupationally- appropriate incentivization or by increasing perceived social and institutional support for mental health monitoring and early intervention.

## 5 Conclusion

The current results guide future explorations of volitional participation and engagement, as well as the development of adherence promoting interventions that consider the pre-existing mental health status of cadets. The multidimensional relationships between Superusers, Contributors, and Lurkers regarding volitional participation in daily surveys during CTP highlights that RCMP Cadet participation does not differ from the general population (17, 42, 43, 63). Lastly, cadets interact with and access the self-monitoring resources and digital social health tools in the same patterns as the general population (17, 18, 42, 43). Evidence that Cadets exhibit interaction patterns similar to the general population, despite reduced barriers to access self-monitoring resources, provides a basis for making generalizations in future analyses. The under-studied longitudinal links between protective variables and mental health may contributed to the diverse research results associated with the limited data available regarding assessments of proactive mental health programs for PSP (64). Creating highly individualized, population based PTSI mitigation programs may also be hampered by insufficient longitudinal data collections as well as disproportionate rates of attrition and disengagement among the very PSP such programs are designed to help. Better understanding patterns of participation in mental health monitoring may also help to improve program effectiveness.

## Data availability statement

The datasets presented in this article are not readily available because of the sensitive nature of the content. Requests to access the datasets should be directed to nick.carleton@uregina.ca.

## Ethics statement

The RCMP Study was approved by the University of Regina Institutional Research Ethics Board (file No. 2019–055) and the RCMP Research Ethics Board (file No. SKM_C30818021312580). The RCMP Study was also approved through a Privacy Impact Assessment as part of the overall approval including the National Administrative Records Management System (NARMS; file No. 201611123286) and Public Services and Procurement Canada (PSPC; file No. 201701491/M7594174191). The studies were conducted in accordance with the local legislation and institutional requirements. The participants provided their written informed consent to participate in this study.

## Author contributions

TT: Conceptualization, Methodology, Validation, Formal analysis, Investigation, Data curation, Writing – original draft, Writing – review & editing. RS: Conceptualization, Writing – original draft, Writing – review & editing. KA: Conceptualization, Writing – original draft, Writing – review & editing. KM: Conceptualization, Methodology, Validation, Data curation, Writing – original draft, Writing – review & editing. LJ: Conceptualization, Methodology, Data curation, Writing – original draft, Writing – review & editing. JN: Writing – review & editing. TA: Writing – review & editing. LL: Methodology, Writing – review & editing. SS: Writing – review & editing. SS-Z: Investigation, Resources, Writing – review & editing. RK: Writing – review & editing. JN: Investigation, Data curation, Writing – review & editing. GK: Methodology, Validation, Investigation, Resources, Data curation, Writing – review & editing. RC: Conceptualization, Methodology, Validation, Formal analysis, Investigation, Resources, Data curation, Writing – original draft, Writing – review & editing.

## Footnotes

1 Carleton, R. N., Teckchandani, T. A., Sauer-Zavala, S., Maguire, K. Q., Fletcher, A. J., Jamshidi, L., et al. (submitted). Mental Health of Royal Canadian Mounted Police Cadets Completing Training. *Journal of Police and Criminal Psychology*.

## References

[1] Heber A Testa V Groll D Ritchie K Tam-Seto L Mulligan A. Glossary of terms: A shared understanding of the common terms used to describe psychological trauma, version 3.0. Health Promot Chronic Dis Prev Can.. (2019) 43(10/11). doi: 10.24095/hpcdp.43.10/11.09 PMC11386910 37991891

[2] Carleton RN Afifi TO Taillieu T Turner S Krakauer R Anderson GS. Exposures to potentially traumatic events among public safety personnel in Canada. Can J Behav Sci. (2019) 51:37–52. doi: 10.1037/cbs0000115

[3] Papazoglou K. Conceptualizing Police Complex Spiral Trauma and its applications in the police field. Traumatology. (2013) 19:196–209. doi: 10.1177/1534765612466151

[4] Carleton RN Afifi TO Turner S Taillieu T Duranceau S LeBouthillier DM. Mental disorder symptoms among public safety personnel in Canada. Can J Psychiatry. (2018) 63:54–64. doi: 10.1177/0706743717723825 28845686 PMC5788123

[5] Klimley KE Van Hasselt VB Stripling AM. Posttraumatic stress disorder in police, firefighters, and emergency dispatchers. Aggress Violent Behav. (2018) 43:33–44. doi: 10.1016/j.avb.2018.08.005

[6] Sherwood L Hegarty S Vallières F Hyland P Murphy J Fitzgerald G. Identifying the key risk factors for adverse psychological outcomes among police officers: A systematic literature review. J Trauma Stress. (2019) 32:688–700. doi: 10.1002/jts.22431 31553502

[7] Carleton RN Afifi TO Taillieu T Turner S Mason JE Ricciardelli R. Assessing the relative impact of diverse stressors among public safety personnel. Int J Environ Res Public Health. (2020) 17:1234. doi: 10.3390/ijerph17041234 32075062 PMC7068554

[8] Smith JA Braunack-Mayer A Wittert G Warin M. “It’s sort of like being a detective”: Understanding how Australian men self-monitor their health prior to seeking help. BMC Health Serv Res. (2008) 8:56. doi: 10.1186/1472-6963-8-56 18366631 PMC2288603

[9] Carleton RN Krätzig GP Sauer-Zavala S Neary JP Lix LM Fletcher AJ. The Royal Canadian Mounted Police (RCMP) study: Protocol for a prospective investigation of mental health risk and resilience factors. Health Promot Chronic Dis Prev Can. (2022) 42:319–33. doi: 10.24095/hpcdp.42.8.02 PMC9514212 35993603

[10] Hill CLM Updegraff JA. Mindfulness and its relationship to emotional regulation. Emotion. (2012) 12:81–90. doi: 10.1037/a0026355 22148996

[11] Kauer SD Reid SC Crooke AHD Khor A Hearps SJC Jorm AF. Self-monitoring using mobile phones in the early stages of adolescent depression: Randomized controlled trial. J Med Internet Res. (2012) 14:e67. doi: 10.2196/jmir.1858 22732135 PMC3414872

[12] Jarrett RB Nelson RO. Mechanisms of change in cognitive therapy of depression. Behav Ther. (1987) 18:227–41. doi: 10.1016/S0005-7894(87)80017-5

[13] Ramanan M Billot L Rajbhandari D Myburgh J Finfer S Bellomo R. Does asymmetry in patient recruitment in large critical care trials follow the Pareto principle? Trials. (2020) 21:1–8. doi: 10.1186/s13063-020-04279-1 32370789 PMC7201735

[14] Sitthiyot T Holasut K. A simple method for estimating the Lorenz curve. Humanit Soc Sci Commun. (2021) 8:1–9. doi: 10.1057/s41599-021-00948-x 38617731

[15] Binks M Van Mierlo T. Utilization patterns and user characteristics of an ad libitum Internet weight loss program. J Med Internet Res. (2010) 12:1–5. doi: 10.2196/jmir.1347 PMC2872772 20350926

[16] Martínez-Torres MR Toral SL Barrero F Cortés F. The role of Internet in the development of future software projects. Internet Res. (2010) 20:72–86. doi: 10.1108/10662241011020842

[17] Van Mierlo T Voci S Lee S Fournier R Selby P. Superusers in social networks for smoking cessation: Analysis of demographic characteristics and posting behavior from the canadian cancer society’s smokers’ helpline online and stopsmokingcenter.net. J Med Internet Res. (2012) 14. doi: 10.2196/jmir.1854 PMC3414904 22732103

[18] Van Mierlo T. The 1% rule in four digital health social networks: An observational study. J Med Internet Res. (2014) 16:1–9. doi: 10.2196/jmir.2966 PMC3939180 24496109

[19] van Mierlo T Hyatt D Ching AT. Employing the Gini coefficient to measure participation inequality in treatment-focused Digital Health Social Networks. Netw Model Anal Health Inform Bioinform. (2016) 5:1–10. doi: 10.1007/s13721-016-0140-7 PMC5082574 27840788

[20] Joglekar S Sastry N Coulson NS Taylor SJC Patel A Duschinsky R. How online communities of people with long-term conditions function and evolve: Network analysis of the structure and dynamics of the asthma UK and british lung foundation online communities. J Med Internet Res. (2018) 20. doi: 10.2196/jmir.9952 PMC6060304 29997105

[21] Sanders R. Commentary: The pareto principle: Its use and abuse. J Consum Mark. (1987) 4:47–50. doi: 10.1108/eb008188

[22] Newman MEJ. The structure and function of complex networks. SIAM Rev. (2003) 45:167–256. doi: 10.1137/S003614450342480

[23] Schneider A Von Krogh G Jäger P. What’s coming next? Epistemic curiosity and lurking behavior in online communities. Comput Hum Behav. (2013) 29:293–303. doi: 10.1016/j.chb.2012.09.008

[24] Sciadas G. Unveiling the digital divide. Sci Innov Electron Inf Div. (2002) 23.

[25] Shields RE Teckchandani TA Asmundson GJG Nisbet J Krakauer RL Andrews KL. Daily survey participation and positive changes in mental health symptoms scores among Royal Canadian Mounted Police cadets. Front Psychol. (2023) 14:1145194. doi: 10.3389/fpsyg.2023.1145194 37599763 PMC10437217

[26] Hembroff CC Krätzig G. A 5-year perspective of attrition in relation to employment equity. (2020) (Royal Canadian Mounted Police: Research and Strategic Partnerships).

[27] Weathers FW Litz BT Keane TM Palmieri PA Marx BP Schnurr PP. PTSD checklist for military. Natl Cent PTSD. (2013) 94.

[28] Kroenke K Spitzer RL Williams JB. The PHQ-9: validity of a brief depression severity measure. J Gen Intern Med. (2001) 16:606–13. doi: 10.1046/j.1525-1497.2001.016009606.x PMC1495268 11556941

[29] Shear MK Brown TA Barlow DH Money R Sholomskas DE Woods SW. Multicenter collaborative panic disorder severity scale. Am J Psychiatry. (1997) 154:1571–5. doi: 10.1176/ajp.154.11.1571 9356566

[30] Spitzer RL Kroenke K Williams JBW Löwe B. A brief measure for assessing generalized anxiety disorder: the GAD-7. Arch Intern Med. (2006) 166:1092–7. doi: 10.1001/archinte.166.10.1092 16717171

[31] Carleton RN Collimore KC Asmundson GJG McCabe RE Rowa K Antony MM. Refining and validating the social interaction anxiety scale and the social phobia scale. Depress Anxiety. (2009) 26:E71–81. doi: 10.1002/da.20480 19152346

[32] Saunders JB Aasland OG Babor TF de la Fuente JR Grant M. Development of the Alcohol Use Disorders Identification Test (AUDIT): WHO collaborative project on early detection of persons with harmful alcohol consumption-II. Addiction. (1993) 88:791–804. doi: 10.1111/j.1360-0443.1993.tb02093.x 8329970

[33] American Psychiatric Association. Diagnostic and statistical manual of mental disorders. 5th. Washington, DC: American Psychiatric Association (2013). doi: 10.1176/appi.books.9780890425596

[34] Blevins CA Weathers FW Davis MT Witte TK Domino JL. The Posttraumatic Stress Disorder Checklist for DSM-5 (PCL-5): Development and initial psychometric evaluation. J Trauma Stress. (2015) 28:489–98. doi: 10.1002/jts.22059 26606250

[35] Ashbaugh AR Houle-Johnson S Herbert C El-Hage W Brunet A. Psychometric validation of the english and french versions of the posttraumatic stress disorder checklist for DSM-5 (PCL-5). PloS One. (2016) 11:e0161645. doi: 10.1371/journal.pone.0161645 27723815 PMC5056703

[36] Bovin MJ Marx BP Weathers FW Gallagher MW Rodriguez P Schnurr PP. Psychometric properties of the PTSD Checklist for Diagnostic and Statistical Manual of Mental Disorders-Fifth Edition (PCL-5) in veterans. Psychol Assess. (2016) 28:1379–91. doi: 10.1037/pas0000254 26653052

[37] Manea L Gilbody S McMillan D. A diagnostic meta-analysis of the Patient Health Questionnaire-9 (PHQ-9) algorithm scoring method as a screen for depression. Gen Hosp Psychiatry. (2015) 37:67–75. doi: 10.1016/j.genhosppsych.2014.09.009 25439733

[38] Houck PR Spiegel DA Shear MK Rucci P. Reliability of the self-report version of the Panic Disorder Severity Scale. Depress Anxiety. (2002) 15:183–5. doi: 10.1002/da.10049 12112724

[39] Swinson RP. The GAD-7 scale was accurate for diagnosing generalized anxiety disorder. Evid Based Med. (2006) 11:184. doi: 10.1136/ebm.11.6.184 17213178

[40] Gache P Michaud P Landry U Accietto C Arfaoui S Wenger O. The Alcohol Use Disorders Identification Test (AUDIT) as a screening tool for excessive drinking in primary care: Reliability and validity of a French version. Alcohol Clin Exp Res. (2005) 29:2001–7. doi: 10.1097/01.alc.0000187034.58955.64 16340457

[41] Cohen J. Statistical Power Analysis for the Behavioral Sciences. New York, NY: Routledge (2013). doi: 10.4324/9780203771587

[42] Van Mierlo T Hyatt D Ching AT. Mapping power law distributions in digital health social networks: Methods, interpretations, and practical implications. J Med Internet Res. (2015) 17:e160. doi: 10.2196/jmir.4297 26111790 PMC4526963

[43] Carron-Arthur B Cunningham JA Griffiths KM. Describing the distribution of engagement in an Internet support group by post frequency: A comparison of the 90–9-1 Principle and Zipf’s Law. Internet Interv. (2014) 1:165–8. doi: 10.1016/j.invent.2014.09.003

[44] Gulliver A Griffiths KM Christensen H. Perceived barriers and facilitators to mental health help-seeking in young people: A systematic review. BMC Psychiatry. (2010) 10:1–9. doi: 10.1186/1471-244X-10-113 21192795 PMC3022639

[45] Bakker D Kazantzis N Rickwood D Rickard N. Mental health smartphone apps: Review and evidence-based recommendations for future developments. JMIR Ment Health. (2016) 3:e4984. doi: 10.2196/mental.4984 PMC4795320 26932350

[46] Ricciardelli R Czarnuch S Afifi TO Taillieu T Carleton RN. Public Safety Personnel’s interpretations of potentially traumatic events. Occup Med Oxf Engl. (2020) 70:155–61. doi: 10.1093/occmed/kqaa007 PMC7252499 32040152

[47] Feldman G Harley R Kerrigan M Jacobo M Fava M. Change in emotional processing during a dialectical behavior therapy-based skills group for major depressive disorder. Behav Res Ther. (2009) 47:316–21. doi: 10.1016/j.brat.2009.01.005 19232571

[48] Başoğlu M Marks IM Sengün S. A prospective study of panic and anxiety in agoraphobia with panic disorder. Br J Psychiatry J Ment Sci. (1992) 160:57–64. doi: 10.1192/bjp.160.1.57 1347481

[49] Kazantzis N Deane FP Ronan KR L’Abate L. Using homework assignments in cognitive behavior therapy. New York, NY: Taylor and Francis (2005). doi: 10.4324/9780203499825

[50] Cohen JS Edmunds JM Brodman DM Benjamin CL Kendall PC. Using self-monitoring: implementation of collaborative empiricism in cognitive-behavioral therapy. Cogn Behav Pract. (2013) 20:419–28. doi: 10.1016/j.cbpra.2012.06.002

[51] Ehlers A Clark DM Hackmann A McManus F Fennell M Herbert C. A randomized controlled trial of cognitive therapy, a self-help booklet, and repeated assessments as early interventions for posttraumatic stress disorder. Arch Gen Psychiatry. (2003) 60:1024–32. doi: 10.1001/archpsyc.60.10.1024 14557148

[52] Smith BW Dalen J Wiggins K Tooley E Christopher P Bernard J. The brief resilience scale: assessing the ability to bounce back. Int J Behav Med. (2008) 15:194–200. doi: 10.1080/10705500802222972 18696313

[53] Suveg C Hoffman B Zeman JL Thomassin K. Common and specific emotion-related predictors of anxious and depressive symptoms in youth. Child Psychiatry Hum Dev. (2009) 40:223–39. doi: 10.1007/s10578-008-0121-x 19039662

[54] Barrett LF Gross J Christensen TC Benvenuto M. Knowing what you’re feeling and knowing what to do about it: Mapping the relation between emotion differentiation and emotion regulation. Cogn Emot. (2001) 15:713–24. doi: 10.1080/02699930143000239

[55] O’Toole MS Jensen MB Fentz HN Zachariae R Hougaard E. Emotion differentiation and emotion regulation in high and low socially anxious individuals: An experience-sampling study. Cogn Ther Res. (2014) 38:428–38. doi: 10.1007/s10608-014-9611-2

[56] Huppert JD Ledley DR Foa EB. The use of homework in behavior therapy for anxiety disorders. J Psychother Integr. (2006) 16:128–39. doi: 10.1037/1053-0479.16.2.128

[57] Tarrier N Sommerfield C Reynolds M Pilgrim H. Symptom self-monitoring in the treatment of posttraumatic stress disorder. Behav Ther. (1999) 30:597–605. doi: 10.1016/S0005-7894(99)80027-6

[58] Kavanagh DJ Sitharthan T Spilsbury G Vignaendra S. An evaluation of brief correspondence programs for problem drinkers. Behav Ther. (1999) 30:641–56. doi: 10.1016/S0005-7894(99)80030-6

[59] Hoge CW Lesikar SE Guevara R Lange J Brundage JF Engel CC. Mental disorders among U.S. military personnel in the 1990s: Association with high levels of health care utilization and early military attrition. Am J Psychiatry. (2002) 159:1576–83. doi: 10.1176/appi.ajp.159.9.1576 12202280

[60] Ireland CRR Kress AM Frost LZ. Association between mental health conditions diagnosed during initial eligibility for military health care benefits and subsequent deployment, attrition, and death by suicide among active duty service members. Mil Med. (2012) 177:1149–56. doi: 10.7205/MILMED-D-12-00051 23113440

[61] Garcia SMS Ortman BV Burnett DG. Mental health diagnoses and attrition in air force recruits. Mil Med. (2015) 180:436–44. doi: 10.7205/MILMED-D-14-00311 25826349

[62] Andersen JP Di Nota PM Alavi N Anderson G Bennell C McGregor C. A Biological Approach to Building Resilience and Wellness Capacity among police exposed to posttraumatic stress injuries: Protocol for a Randomized Controlled Trial. JMIR Res Protoc. (2023) 12:e33492. doi: 10.2196/33492 37223981 PMC10248782

[63] Jennings C Lhuede K Bradley G Pepin G Hitch D. Activity participation patterns of community mental health consumers. Br J Occup Ther. (2021) 84:561–70. doi: 10.1177/0308022620945166

[64] Di Nota PM Bahji A Groll D Carleton RN Anderson GS. Proactive psychological programs designed to mitigate posttraumatic stress injuries among at-risk workers: a systematic review and meta-analysis. Syst Rev. (2021) 10:126. doi: 10.1186/s13643-021-01677-7 33910641 PMC8079856

